# Treatment of Scarlet Fever

**Published:** 1872-11

**Authors:** 


Treatment of Scarlet Fever.—The late Prof. Geo. T. Elliot in a lecture on this disease, gave the following method of treatment: To bring the eruption out, if it has not already presented itself, order hot boths and blankets. Give nothing to eat at first in the eruptive stage, and only allow the simplest nourishment the first day. Patients experience great relief from baths, and the application of cold cream, or mutton tallow, over the whole body. Visit the patient twice a day. By pouring a pitcherful of cold water over the back of the neck, especially when the glands are enlarged, great comfort is experienced. As a gargle, make use of chlorate of potash or soda. Pieces of ice are good, in the mouth. Sprays, thrown in with Richardson's instrument, of lime water, solutions of alum and sulphate of zinc, are beneficial. As a palliative to the throat, the vapor from slaked lime can be recommended. Strong beef tea, with opium, may be thrown up the bowel. Begin to feed the patient from the second day of the eruption with animal essences. If the tonsils are enlarging and the pharynx exhibits much redness, with diphtheritic exudation, the physician has a right to say that things look bad. If the throat symptoms do not mitigate on the fourth or fifth day, the voice being affected, then one feels that there is a good deal of danger. When the kidneys show hyperæmia, then there is a twofold danger. Always examine the urine. When the patient has kidney disease, the treatment should be directed to the skin and bowels; when the latter are loaded and constipated, give powerful saline cathartics. Get Ronochetti's apparatus, to produce perspiration. To convalescing patients the use of iron is beneficial. The bisulphites have been recommended, but, from experience, they cannot be advocated. Belladonna is not always a prophylactic, although, on account of its innocence, and a feeling of satisfaction to the practitioner and family, it is well to administer it.—*Medical Record.*

